# Clinical and Biochemical Characteristics of Pseudohypoaldosteronism Type 1 with and Without Genetic Mutations: A Literature Review

**DOI:** 10.3390/jcm14134408

**Published:** 2025-06-20

**Authors:** Yuki Nakata, China Nagano, Yukihito Imagawa, Keisuke Shirai, Yu Masuda, Takumi Kido, Mariko Ashina, Kandai Nozu, Kazumichi Fujioka

**Affiliations:** Department of Pediatrics, Kobe University Graduate School of Medicine, Kobe 650-0017, Japan; yuki111@med.kobe-u.ac.jp (Y.N.); china@med.kobe-u.ac.jp (C.N.); yuki0205@med.kobe-u.ac.jp (Y.I.); ksk1024@med.kobe-u.ac.jp (K.S.); yumasuda@med.kobe-u.ac.jp (Y.M.); tkido@med.kobe-u.ac.jp (T.K.); marikoa@med.kobe-u.ac.jp (M.A.); nozu@med.kobe-u.ac.jp (K.N.)

**Keywords:** pseudohypoaldosteronism, renin, aldosterone, hyperkalemia, hyponatremia, mineralocorticoid receptor, epithelial sodium channel, MR, ENaC

## Abstract

**Background/Objectives:** Pseudohypoaldosteronism type 1 (PHA-1) is a rare disorder characterized by aldosterone resistance, leading to hyponatremia, hyperkalemia, and elevated renin and aldosterone levels in neonates and infants. While genetic mutations in *NR3C2* (mineralocorticoid receptor, MR) and *SCNN1A/B/G* (epithelial sodium channel, ENaC) are established causes of primary PHA-1, cases without detectable mutations have also been reported. This study aimed to compare the clinical characteristics of genetically confirmed PHA-1 cases—with or without mutations—and to assess genotype–phenotype correlations. **Methods:** A literature review was conducted using the Medline database, covering studies published from 1966 to October 2023. Included cases were diagnosed with PHA-1 and had undergone genetic testing for *NR3C2* and *SCNN1A/B/G*. Clinical and biochemical data were compared across three groups: MR, ENaC, and non-mutation. Additional subgroup analysis based on mutation type (truncating vs. non-truncating) was also performed. **Results:** A total of 164 patients from 64 studies met the inclusion criteria. The ENaC group showed significantly higher serum potassium levels than the MR and non-mutation groups. Serum aldosterone levels were significantly higher in the MR group compared to the non-mutation group. A genotype–phenotype correlation was evident in the ENaC group, with truncating variants associated with more severe hyperkalemia. No such correlation was observed in the MR group. **Conclusions:** This review highlights distinct clinical features of PHA-1 according to genetic status. Aldosterone levels may aid in guiding decisions regarding genetic testing. Furthermore, variant type in ENaC-related PHA-1 may predict biochemical severity and should be considered in clinical management strategies.

## 1. Introduction

Pseudohypoaldosteronism type 1 (PHA-1) is a rare disorder caused by aldosterone resistance in the renal tubules, leading to impaired sodium reabsorption. It typically presents in neonates or infants with hyponatremia, hyperkalemia, elevated renin and aldosterone levels, and occasional metabolic acidosis [1,2]. Despite normal renal glomerular and adrenal function, patients may experience a range of clinical symptoms, from relatively mild lethargy and poor feeding to severe, life-threatening conditions such as arrhythmias and shock [3,4].

PHA-1 is distinct from PHA-2, a disorder involving increased sodium and chloride reabsorption in the distal tubules. PHA-2 is more commonly seen in adults and is characterized by hypertension, hyperkalemia due to fluid overload, and variable aldosterone levels with suppressed renin, reflecting a different pathophysiology [5].

PHA-1 is classified as either primary or secondary. Primary PHA-1 results from pathogenic mutations in causative genes and is further categorized into two forms: renal, due to mutations in the *NR3C2* gene encoding the mineralocorticoid receptor (MR), and systemic, due to mutations in *SCNN1A*, *SCNN1B*, or *SCNN1G*, which encode subunits of the epithelial sodium channel (ENaC) [6]. Secondary PHA-1 arises from non-genetic causes such as urinary tract infections or congenital abnormalities and typically resolves with treatment of the underlying condition [6,7].

The clinical course of PHA-1 varies. The renal form is usually milder and often no longer requires sodium chloride (NaCl) supplementation by early childhood (around 3 years of age), whereas the systemic form is more severe and may require lifelong NaCl supplementation [2,6]. However, exceptions exist: some systemic cases improve within months [8], while some renal cases can present with severe, life-threatening symptoms [9], indicating a wide clinical spectrum.

Secondary PHA-1 generally manifests between the neonatal period and late infancy [6,7], with most cases resolving within 1–2 weeks of treating the underlying infection [7]. However, prolonged courses can occur in cases involving renal parenchymal damage or persistent urinary tract anomalies [7,10].

Experimental studies suggest that aldosterone resistance may be physiological in neonates. For instance, MR and ENaC expressions in mice decline shortly after birth, while in humans, MR expression decreases between 30 and 40 weeks of gestation [11]. Moreover, neonatal blood (cord blood) exhibits higher potassium, renin, and aldosterone levels than maternal blood [12]. These findings indirectly suggest physiological aldosterone resistance in neonatal renal tubules, supporting a hypothesis of transient aldosterone resistance in early life.

A recent Japanese nationwide surveillance study (2019–2021) reported four cases each of primary and secondary PHA-1, along with seven unclassified cases, eight of which showed rapid improvement within 30 days [13]. These findings suggest the existence of transient forms of PHA-1 that do not fit conventional categories and may go undiagnosed.

To better understand these patterns, it is essential to examine cases with confirmed genetic testing. This study presents a review of the literature on PHA-1 to clarify the clinical characteristics and genotype–phenotype relationships in cases with and without identifiable mutations.

## 2. Materials and Methods

This study included patients diagnosed with PHA-1 who underwent genetic testing for known causative genes. A literature search was conducted using the MEDLINE database via the International Medical Information Center Foundation, covering publications from 1966 through 19 October 2023. The search strategy employed a combination of Medical Subject Headings (MeSHs) and free-text keywords related to PHA-1 and its genetic and clinical features. The complete search strategy is detailed in Supplementary Table S1.

Exclusion criteria encompassed non-English articles, studies lacking individual case data, cases that did not meet diagnostic criteria for PHA-1, absence of genetic testing, or missing data on serum renin activity/concentration or aldosterone concentration. Duplicate reports were also excluded. Additional relevant cases identified through a manual review of references were included.

Eligible cases were categorized into three groups based on genetic findings: (1) MR group: patients with mutations in *NR3C2* (OMIM* 600983); (2) ENaC group: patients with mutations in *SCNN1A* (OMIM* 600228), *SCNN1B* (OMIM* 600760), or *SCNN1G* (OMIM* 600761); and (3) non-mutation group: patients with no identifiable mutations in *NR3C2* or the ENaC genes. Extracted data included sex, gestational age, age at presentation or diagnosis, identified mutations, serum sodium and potassium levels, aldosterone concentration, renin activity or concentration, presence of secondary factors (e.g., urinary tract anomalies), and the requirement for sodium supplementation.

Furthermore, for the MR and ENaC groups, genetic variants were classified as follows: (a) truncating variants: nonsense or frameshift variants; and (b) non-truncating variants: missense or in-frame mutations. Intronic variants were assessed using SpliceAI, with a score ≥ 0.2 considered indicative of potential splicing alterations [14]. In the ENaC group, compound heterozygotes harboring both truncating and non-truncating mutations were classified as truncating. In addition, cases in which the mutation was identified but the specific variant details were unavailable were excluded from the comparison between truncating and non-truncating variant groups.

Statistical analyses were performed using the Mann–Whitney U test for between-group comparisons and the Kruskal–Wallis test for comparisons among the three groups. Categorical variables were analyzed using Fisher’s exact test. Statistical significance was set at *p* < 0.05.

## 3. Results

### 3.1. Study Subsection

**Figure 1:**
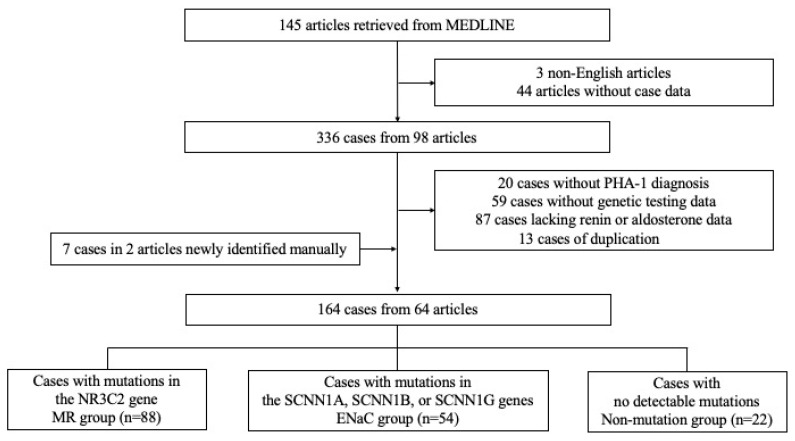
Participant flow diagram.

A total of 145 articles were initially identified. After screening, three non-English articles and 44 articles without case-level data (e.g., reviews, cell/animal studies) were excluded. Of the remaining 98 articles (comprising 336 cases), 20 were excluded due to another diagnosis, 59 lacked genetic testing data, 87 had missing serum renin activity/concentration or aldosterone data, and 13 were duplicate reports. An additional seven cases were identified through manual reference review. Ultimately, 64 articles comprising 164 cases were included in the final analysis (Figure 1).

### 3.2. Comparison Among MR, ENaC, and Non-Mutation Groups

Of the 164 genetically tested PHA-1 cases, 88 (53.7%) were classified into the MR group, 54 (32.9%) into the ENaC group, and 22 (13.4%) into the non-mutation group. Male patients accounted for 53% (*n* = 47) of the MR group, 43% (*n* = 23) of the ENaC group, and 50% (*n* = 11) of the non-mutation group. The differences were not statistically significant (*p* = 0.45). The proportions of preterm infants (gestational age < 37 weeks) were 9% (*n* = 8) in the MR group, 11% (*n* = 6) in the ENaC group, and 9% (*n* = 2) in the non-mutation group, with no significant differences observed (*p* = 0.93). The presence of secondary factors was reported in six (7%) MR cases, three (6%) ENaC cases, and three (14%) non-mutation cases, with no significant differences observed (*p* = 0.49). Secondary factors included urinary tract infection (*n* = 3), hydronephrosis (*n* = 4), renal hemorrhage (*n* = 1), nephroptosis (*n* = 1), fetal renal hypertrophy (*n* = 1), intestinal perforation (*n* = 1), and enteritis (*n* = 1) (Table 1).

The presentation of PHA-1 during the neonatal period (within 28 days of birth) was observed in 57 cases (65%) in the MR group, 45 cases (83%) in the ENaC group, and 13 cases (59%) in the non-mutation group. This difference was statistically significant (*p* = 0.03), with the ENaC group showing a significantly higher neonatal presentation rate compared to the MR and non-mutation groups (*p* = 0.02, 0.04, respectively).

When considering presentation within the first 3 months of life, 72 cases (82%) in the MR group, 53 cases (98%) in the ENaC group, and 18 cases (82%) in the non-mutation group met this criterion. The difference among groups was significant (*p* < 0.01), with the ENaC group having a significantly higher early presentation rate than both the MR (*p* < 0.01) and non-mutation groups (*p* < 0.05) (Table 1).

Plasma renin levels exceeded the upper measurement limit in 36 patients (22%), and 1 case lacked unit information. Among the analyzable data, plasma renin activity (PRA) was available for 80 cases, with a median of 82.82 ng/mL/h (range: 0.5–6,410,000), and active renin concentration (ARC) for 47 cases, with a median of 1047 pg/mL (range: 6.6–323,000). PRA levels did not differ significantly among the three groups (*p* = 0.14), while ARC levels did (*p* < 0.01), with the MR group showing significantly higher ARC than both the ENaC (*p* < 0.05) and non-mutation groups (*p* < 0.01).

Median serum sodium levels were significantly different among groups (*p* < 0.01): 127.5 mEq/L (range: 113.3–140.0) in the MR group (*n* = 82), 124.0 mEq/L (105.0–135.0) in the ENaC group (*n* = 54), and 126.0 mEq/L (114.3–134.0) in the non-mutation group (*n* = 21). The ENaC group had significantly lower sodium levels than the MR group (*p* < 0.01).

Serum potassium levels also differed significantly (*p* < 0.0001): median values were 6.3 mEq/L (range: 4.9–10.5; *n* = 79) in the MR group, 9.0 mEq/L (5.1–13.0; *n* = 53) in the ENaC group, and 6.53 mEq/L (4.9–11.4; *n* = 21) in the non-mutation groups. The ENaC group exhibited significantly higher potassium levels than both the MR and non-mutation groups (*p* < 0.001).

Median serum aldosterone levels were 9170 pg/mL (range: 368.0–45,600; *n* = 72) in the MR group, 6440 pg/mL (209.1–45,745; *n* = 48) in the ENaC group, and 3585 pg/mL (234.3–34,402; *n* = 22) in the non-mutation group. These differences were significant (*p* = 0.02), with the MR group having higher aldosterone levels than the non-mutation group (*p* < 0.01).

We also analyzed the aldosterone/renin ratio (ARR), which is commonly used to diagnose primary aldosteronism. Using plasma renin activity (PRA), ARR could be calculated in 36 cases in the MR group, with a median value of 289.8 pmol/L per ng/mL/h (range: 0.0–39,104.5); in 26 cases in the ENaC group, with a median of 188.2 pmol/L per ng/mL/h (range: 0.2–1924.5); and in 11 cases in the non-mutation group, with a median of 300.0 pmol/L per ng/mL/h (range: 60.0–5548.0). There was no significant difference among the three groups (*p* = 0.28). The number of cases exceeding the threshold of 750 pmol/L per ng/mL/h, a proposed cutoff for predicting primary aldosteronism [16], was 8/36 (22.2%) in the MR group, 1/26 (3.8%) in the ENaC group, and 2/11 (18.1%) in the non-mutation group. Using active renin concentration (ARC), ARR could be calculated in 18 cases in the MR group, with a median value of 9.8 pmol/ng (range: 0.1–148.7); in 15 cases in the ENaC group, with a median of 33.8 pmol/ng (range: 0.1–302.9); and in 9 cases in the non-mutation group, with a median of 98.5 pmol/ng (range: 0.1–1131.7). There was no significant difference among the three groups (*p* = 0.06). The number of cases exceeding the threshold of 150 pmol/ng, a proposed cutoff for predicting primary aldosteronism [16], was 0/18 (0%) in the MR group, 3/15 (20%) in the ENaC group, and 3/9 (33.3%) in the non-mutation group.

The proportion of patients requiring sodium supplementation was 92% (*n* = 81) in the MR group, 96% (*n* = 52) in the ENaC group, and 86% (*n* = 19) in the non-mutation group. However, these differences were not statistically significant (*p* = 0.25).

### 3.3. Comparison Between Truncating and Non-Truncating Variants

Among the 88 cases in the MR group, 64 had truncating variants, 19 had non-truncating variants, and 5 had variants that were either not reported or could not be classified (Supplementary Table S2). Among the 54 cases in the ENaC group, 37 had truncating variants (including 3 cases with compound heterozygous truncating and non-truncating variants), 15 had non-truncating variants, and 2 had variants that were either not reported or could not be classified (Supplementary Table S3). In the MR group, no significant differences were found between truncating and non-truncating variants in terms of serum sodium: 127 mEq/L (range: 113.3–140, *n* = 60) vs. 128 mEq/L (range: 114–136, *n* = 17; *p* = 0.76); serum potassium: 6.3 mEq/L (5.1–10.5, *n* = 60) vs. 6.7 mEq/L (4.9–9.7, *n* = 14; *p* = 0.14); and aldosterone: 9170 pg/mL (1153–45,600, *n* = 54) vs. 7592 pg/mL (368–38,530, *n* = 14; *p* = 0.91). However, PRA was significantly higher in the non-truncating group (200 ng/mL/h [65.4–15,176, *n* = 9]) than in the truncating group (74 ng/mL/h [6.858–2,531,400, *n* = 31]; *p* = 0.04). No significant difference in ARC was observed (3000 pg/mL [101–322,899, *n* = 15] vs. 1460 pg/mL [1266–52,668, *n* = 5]; *p* = 0.67). Among cases in which renin was measured using plasma renin activity (PRA), ARR could be calculated in 27 cases in the truncating variant group, with a median of 321.6 pmol/L per ng/mL/h (range: 0.0–4674.2), and in 8 cases in the non-truncating group, with a median of 72.9 pmol/L per ng/mL/h (range: 1.9–1083.8). There was no significant difference between the two groups (*p* = 0.24). Among cases in which renin was measured using active renin concentration (ARC), ARR could be calculated in 13 cases in the truncating group, with a median of 5.0 pmol/ng (range: 0.1–78.3), and in 3 cases in the non-truncating group, with a median of 11.0 pmol/ng (range: 8.6–21.9). There was no significant difference between the two groups (*p* = 0.61).

In the ENaC group, no significant differences were found in serum sodium levels between truncating (123 mEq/L [105–135, *n* = 37]) and non-truncating (125 mEq/L [106–133.4, *n* = 15]; *p* = 0.18) variants. However, serum potassium was significantly higher in truncating variants (9.3 mEq/L [5.1–13, *n* = 36]) compared to non-truncating variants (7.7 mEq/L [5.4–10, *n* = 15]; *p* < 0.01). No significant differences were found in aldosterone levels (6168 pg/mL [209.1–45,745, *n* = 32] vs. 8560 pg/mL [806–20,880, *n* = 14]; *p* = 0.46), or in PRA (84.5 ng/mL/h [5.36–192,000, *n* = 22] vs. 140 ng/mL/h [13.39–6,410,000, *n* = 3]; *p* = 0.78). However, ARC was significantly higher in the truncating group (1092 pg/mL [160.3–104,200, *n* = 8]) than in the non-truncating group (187.1 pg/mL [21–96,900, *n* = 8]; *p* < 0.05) (Table 2). Among cases in which renin was measured using plasma renin activity (PRA), ARR could be calculated in 22 cases in the truncating variant group, with a median of 259.0 pmol/L per ng/mL/h (range: 0.2–1924.5), and in 2 cases in the non-truncating group, with a median of 428.7 pmol/L per ng/mL/h (range: 187.4–669.9). There was no significant difference between the two groups (*p* = 0.59). Among cases in which renin was measured using active renin concentration (ARC), ARR could be calculated in seven cases in the truncating group, with a median of 21.0 pmol/ng (range: 0.1–184.3), and in eight cases in the non-truncating group, with a median of 95.5 pmol/ng (range: 0.1–302.9). There was no significant difference between the two groups (*p* = 0.15).

The unit of ARC was converted using the formula: μIU/mL × 0.6 = pg/mL [15]. Data are presented as medians (range, numbers). Na, sodium; K, potassium; PRA, plasma renin activity; ARC, active renin concentration.

## 4. Discussion

In this literature review of genetically confirmed PHA-1 cases, we found that patients in the ENaC group exhibited significantly lower serum sodium levels than in the MR group, while higher potassium levels than in both MR and non-mutation groups. Serum aldosterone concentrations were higher in the MR group than in the non-mutation group. While no clear genotype–phenotype correlation was observed in the MR group, a notable association was identified in the ENaC group, where truncating variants were linked to more severe hyperkalemia than non-truncating variants.

This study aimed to compare the clinical and biochemical characteristics of three genetically defined groups: MR, ENaC, and non-mutation. Previous studies, such as that by Adachi et al. [17], reported more pronounced hyponatremia and hyperkalemia in patients with ENaC mutations than those with MR mutations. However, those studies did not include cases lacking identified genetic mutations and often omitted data on serum aldosterone levels. Our analysis not only supports earlier findings regarding electrolyte imbalances in the ENaC group but also contributes new insights—most notably, that serum aldosterone concentrations were highest in the MR group. Interestingly, the non-mutation group exhibited hyperkalemia comparable to that in the MR group but with significantly lower aldosterone levels. In the non-mutation group, the presence of hyperkalemia comparable to that seen in the MR group with known mutations may be attributed to insufficient sodium supplementation or physiological aldosterone resistance during the neonatal period. Although the proportion of cases receiving sodium supplementation did not differ among the three groups in this study, detailed comparisons regarding the dosage and duration of supplementation were not feasible. This remains an important issue for future investigation. We also conducted an analysis using the aldosterone/renin ratio (ARR), but found no significant differences among the three groups. In primary aldosteronism, excessive aldosterone secretion enhances sodium reabsorption, raises blood pressure, and suppresses renin release via negative feedback [18]. In contrast, in PHA-1, resistance to mineralocorticoids in the kidneys and other tissues results in elevated levels of both plasma renin and aldosterone [19]. Given the fundamentally different mechanisms of renin and aldosterone regulation in primary aldosteronism and PHA-1, it is reasonable that ARR did not prove useful for the diagnosis of PHA-1 in the present study. Although transient secondary PHA-1 has been described in earlier reports, genetic testing was rarely performed in those cases [7,20]. To our knowledge, this is the first study to compare cases without identified mutations to those with confirmed *NR3C2* or *SCNN1A/B/G* mutations, providing a broader understanding of the biochemical spectrum of PHA-1.

It is well known that patients in the ENaC group tend to exhibit more severe electrolyte abnormalities, more pronounced clinical symptoms, and a longer symptomatic period compared to those in the MR group. Our findings, showing more marked hyponatremia and hyperkalemia in the ENaC group, are consistent with these previous observations.

The mineralocorticoid receptor (MR), which is encoded by the NR3C2 gene, is a nuclear receptor expressed in various tissues, including the distal renal tubules, airways, sweat glands, cardiovascular system, and central nervous system. It forms a complex with aldosterone and functions as a transcription factor. In the kidney, MR regulates sodium reabsorption and potassium excretion through channels such as ROMK and ENaC; therefore, in renal-type PHA-1, symptoms are primarily limited to the kidneys [2,6].

NR3C2 mutations follow an autosomal dominant inheritance pattern, leading to either loss of function or functionally impaired MR proteins. The pathogenic mechanisms are thought to involve haploinsufficiency or dominant-negative effects caused by MR proteins with pathogenic variants [2]. Clinical improvement with age is often observed, which may be due to an age-related increase in MR expression that compensates for haploinsufficiency [21], as well as the maturation of sodium-retaining capacity, allowing more proximal segments of the nephron to contribute to sodium reabsorption [22].

The epithelial sodium channel (ENaC) is a membrane-bound ion channel selectively permeable to sodium and is composed of a heterotrimer of three homologous subunits: α, β, and γ. It is expressed in the distal renal tubules, collecting ducts, colon, salivary glands, sweat glands, and lungs [2,6,23,24]. In systemic PHA-1, salt loss from these organs leads to generalized symptoms such as dehydration, miliaria rubra (heat rash), and recurrent respiratory infections [2,6]. The genes encoding the α, β, and γ subunits—SCNN1A, SCNN1B, and SCNN1G, respectively—follow an autosomal recessive inheritance pattern, and heterozygous carriers are typically asymptomatic [23].

Among the ENaC-related forms of PHA-1, SCNN1A mutations are the most frequently reported [23]. However, to date, no studies have conclusively demonstrated whether mutations in different ENaC subunits lead to distinct clinical phenotypes.

In our study, the number of cases for each subtype (SCNN1A/B/G) was insufficient to allow for a meaningful comparison of clinical features, but this remains an important area for future investigation.

The non-mutation group was analyzed as a population that does not necessarily correspond to typical secondary PHA-1. Among the 164 cases included in the study, secondary causes were identified in only 12. The proportion was 7% in the MR group, 6% in the ENaC group, and 14% in the non-mutation group, indicating that the frequency of secondary causes was not markedly higher in the non-mutation group. Accordingly, we considered it inappropriate to define this entire group as secondary PHA-1. This group may include patients with unknown genetic causes or transient physiological aldosterone resistance, particularly in the perinatal period, which can present with a phenotype resembling PHA-1 [11,12,13]. By comparing the clinical characteristics of the non-mutation group with those of the MR and ENaC groups, we hope to provide useful reference data to support clinical decision-making regarding genetic testing.

As part of the genotype–phenotype correlation analysis, we compared the truncating and non-truncating variant groups. In the MR group, no genotype–phenotype correlation was observed for sodium, potassium, or aldosterone levels. In contrast, in the ENaC group, a significant genotype–phenotype correlation was found for potassium, with higher levels observed in the truncating variant group. Regarding aldosterone levels, a previous report indicated that patients with missense variants in the ENaC group had lower levels than those with nonsense variants [23]. However, in our study, there was no significant difference in aldosterone levels between the truncating and non-truncating groups within the ENaC cohort, yielding a different result. Given the larger sample size in our study, the findings may be more reliable. As for renin, PRA showed a difference only in the MR group, while ARC showed a difference only in the ENaC group, resulting in inconsistent findings.

Several studies have suggested that nonsense, frameshift, and aberrant splice-site variants are associated with more severe phenotypes than missense mutations [25,26]. In a review by Edelheit et al. involving 22 previously reported cases of systemic PHA-1, 12 of 19 patients with nonsense, frameshift, or splice-site mutations exhibited severe phenotypes. In contrast, among the three patients with missense variants, two with available clinical data presented with mild phenotypes. Based on these findings, the authors concluded that mutations preventing the production of full-length ENaC proteins tend to be associated with more severe disease, whereas missense mutations are generally associated with milder presentations [26]. Similarly, Hanukoglu et al. performed a functional expression analysis using *Xenopus laevis* oocytes to assess ENaC activity in four patients with systemic PHA-1. In a compound heterozygous case carrying a missense mutation, ENaC activity was approximately 40% of that observed in wild-type channels, whereas a homozygous nonsense mutation resulted in less than 5% activity. These functional differences are thought to underlie the observed variations in biochemical parameters and clinical severity [25].

Furthermore, the severity of hyperkalemia was significantly greater in the truncating variant group than in the non-truncating variant group. In vitro studies have demonstrated that certain nonsense mutations result in lower ENaC activity compared to specific missense mutations [25], suggesting that truncating variants may lead to greater impairment of ENaC function than non-truncating variants. However, although serum potassium levels were statistically higher in the truncating group, there was substantial overlap in the values, making it difficult to conclude that this finding is clinically meaningful. Several factors may explain the wide range of values observed. First, it is unclear whether the measurements were taken at the peak of electrolyte imbalance. Second, functional differences may exist depending on the extent to which the ENaC protein is truncated. Additionally, previous studies have shown that expression of the Na-Cl cotransporter (NCC) in the distal tubules is upregulated in ENaC-related PHA-1 [6], potentially compensating for reduced ENaC function. The degree of this compensatory mechanism may vary among individuals. The discrepancy in renin levels between the truncating and non-truncating groups across PRA and ARC measurements may be attributed to the limited number of analyzable cases due to the high rate of data exclusion and the need to evaluate different assay systems separately. Additionally, there was no significant difference in the aldosterone/renin ratio (ARR) between the truncating and non-truncating groups. As ARR is an indicator for primary aldosteronism, it did not show any clear association with sodium, potassium, or other values in the present analysis of clinically diagnosed PHA-1 cases.

Based on our findings, serum aldosterone levels may serve as a useful indicator for determining the need for genetic testing in suspected PHA-1 cases. In the non-mutation group (cases without identified genetic variants), the median aldosterone concentration was 3585 pg/mL (234.3–33,402; *n* = 22), whereas in the mutation group (including both MR and ENaC variants), the median was significantly higher at 7695 pg/mL (209.1–45,745; *n* = 120). No significant differences were observed between the groups in terms of sodium, potassium, or renin levels. These results suggest the potential for developing a diagnostic algorithm for suspected PHA-1 cases that incorporates aldosterone levels to guide the decisions on whether to pursue genetic testing.

This study has some limitations. First, as a literature-based review, many cases had missing data, and it was often unclear whether biochemical evaluations were performed during the peak of electrolyte imbalance. Although significant intergroup differences were observed, the values exhibited broad ranges with considerable overlap. Standardizing the timing of biochemical sample collection in future studies may help yield more accurate and interpretable data. Second, clinical severity was difficult to assess due to inconsistent descriptions, and secondary contributing factors could not always be ruled out. Third, variability in renin assay methods limited the ability to directly compare PRA and ARC. Fourth, there are no universally accepted diagnostic criteria for PHA-1, and the inclusion of cases depends on individual study definitions. Consequently, case inclusion was based on the judgment of each study’s authors, leading to potential inconsistencies. Future research should focus on prospective data collection using standardized diagnostic criteria and measurement protocols to enhance the accuracy and reliability of clinical and biochemical comparisons. Finally, although our review followed a structured and comprehensive search strategy, we did not adopt a formal systematic review methodology. This was due to the inherent heterogeneity of the published data, the predominance of individual case reports, and the lack of standardized outcome measures, which made formal bias assessment and meta-analysis infeasible. Instead, we focused on extracting detailed individual-level data to enable subgroup-level comparisons and phenotype–genotype analysis.

## 5. Conclusions

This literature review of genetically confirmed PHA-1 cases demonstrated that patients in the ENaC group experienced more severe hyponatremia than those in the MR group and more pronounced hyperkalemia than both the MR and non-mutation groups. Serum aldosterone concentrations were significantly higher in the MR group compared to the non-mutation group. While no genotype–phenotype correlation was observed in the MR-related PHA-1, a clear association was found in the ENaC group, where truncating variants were linked to more severe hyperkalemia than non-truncating variants. These findings indicate that genetic variant type—particularly in ENaC-related PHA-1—may serve as a valuable indicator of biochemical severity. Moreover, serum aldosterone levels may aid in the diagnostic evaluation of suspected PHA-1 cases and help determine the need for genetic testing. Integrating both genetic and biochemical profiling into clinical workflows could enhance diagnostic accuracy and inform more personalized treatment strategies for PHA-1.

## Figures and Tables

**Table 1:** Patient characteristics among the MR, ENaC, and non-mutation groups.

	MR (*n* = 88)	ENaC (*n* = 54)	Non-Mutation (*n* = 22)	*p*-Value
Male sex (%)	47 (53%)	23 (43%)	11 (50%)	0.45
Preterm birth	8 (9%)	6 (11%)	2 (9%)	0.93
Presence of secondary factors	6 (7%)	3 (6%)	3 (14%)	0.49
Presentation within 28 days after birth ^a,b^	57 (65%)	45(83%)	13 (59%)	0.03
Presentation within 3 months after birth ^a,b^	72 (82%)	53 (98%)	18 (82%)	<0.01
Presentation within 12 months after birth	87 (99%)	54 (100%)	22 (100%)	>0.99
Sodium supplementation	81 (92%)	52 (96%)	19 (86%)	0.25
Serum sodium levels (mEq/L) ^a^	127.5 (113.3–140.0) *n* = 82	124.0 (105.0–135.0) *n* = 54	126.0 (114.3–134.0) *n* = 21	<0.01
Serum potassium levels (mEq/L) ^a,b^	6.3 (4.9–10.5) *n* = 79	9.0 (5.1–13.0) *n* = 53	6.53 (4.9–11.4) *n* = 21	<0.01
Plasma renin activity (PRA, ng/mL/h)	100.2 (3.1–2,531,400) *n* = 42	140 (5.36–6,410,000) *n* = 27	38.8 (0.5–430) *n* = 11	0.14
Active renin concentration (ARC, pg/mL) ^a,c^	2838 (101–323,000) *n* = 22	396 (21–104,200) *n* = 16	116 (6.6–18,000) *n* = 9	<0.01
Serum aldosterone concentrations (pg/mL) ^c^	9170 (368.0–45,600) *n* = 72	6440 (209.1–45,745) *n* = 48	3585 (234.3–34,402) *n* = 22	0.02
Aldosterone/renin ratio (pmol/L per ng/mL/h)	289.8 (0.0–39,104.5) *n* = 36	188.2 (0.2–1924.5) *n* = 26	300.0 (60.0–5548.0) *n* = 11	0.28
Aldosterone/renin ratio (pmol/ng)	9.8 (0.1–148.7) *n* = 18	33.8 (0.1–302.9) *n* = 15	98.5 (0.1–1131.7) *n* = 9	0.06

The unit of ARC was converted using the formula: μIU/mL × 0.6 = pg/mL [15]. Data are presented as medians (range, number) or numbers (%). PRA, plasma renin activity; ARC, active renin concentration. ^a^: *p* < 0.05 between MR and ENaC groups. ^b^: *p* < 0.05 between ENaC and non-mutation groups. ^c^: *p* < 0.05 between MR and non-mutation groups.

**Table 2:** Comparison between truncating and non-truncating groups.

	Truncating	Non-Truncating	*p*-Value
MR Group			
Na (mEq/L)	127 (113.3–140, *n* = 60)	128 (114–136, *n* = 17)	0.76
K (mEq/L)	6.3 (5.1–10.5, *n* = 60)	6.7 (4.9–9.7, *n* = 14)	0.14
Plasma renin activity (PRA, ng/mL/h)	74 (6.858–2,531,400, *n* = 31)	200 (65.4–15,176, *n* = 9)	0.04
Active renin concentration (ARC, pg/mL)	3000 (101–322,899, *n* = 15)	1460 (1266–52,668, *n* = 5)	0.67
Aldosterone (pg/mL)	9170 (1153–45,600, *n* = 54)	7592 (368–38,530, *n* = 14)	0.91
Aldosterone/renin ratio (pmol/L per ng/mL/h)	321.6 (0.0–4674.2, *n* = 27)	72.9 (1.9–1083.8, *n* = 8)	0.24
Aldosterone/renin ratio (pmol/ng)	5.0 (0.1–78.3, *n* = 13)	11.0 (8.6–21.9, *n* = 3)	0.61
ENaC Group			
Na (mEq/L)	123 (105–135, *n* = 37)	125 (106–133.4, *n* = 15)	0.18
K (mEq/L)	9.3 (5.1–13, *n* = 36)	7.7 (5.4–10, *n* = 15)	<0.01
Plasma renin activity (PRA, ng/mL/h)	84.5 (5.36–192,000, *n* = 22)	140 (13.39–6,410,000, *n* = 3)	0.78
Active renin concentration (ARC, pg/mL)	1092 (160.3–104,200, *n* = 8)	187.1 (21–96,900, *n* = 8)	<0.05
Aldosterone (pg/mL)	6168 (209.1–45,745, *n* = 32)	8560 (806–20,880, *n* = 14)	0.46
Aldosterone/renin ratio (pmol/L per ng/mL/h)	259.0 (0.2–1924.5, *n* = 22)	428.7 (187.4–669.9, *n* = 2)	0.59
Aldosterone/renin ratio (pmol/ng)	21.0 (0.1–184.3, *n* = 7)	95.5 (0.1–302.9, *n* = 8)	0.15

## Data Availability

All relevant data are contained within this manuscript.

## Supplementary Materials

The following supporting information can be downloaded at: https://www.mdpi.com/article/10.3390/jcm14134408/s1, Table S1: The full literature search strategy. Table S2: Summary of Reported Cases of Pseudohypoaldosteronism Type 1 with Identified NR3C2 Mutations (MR Group). Table S3: Summary of Reported Cases of Pseudohypoaldosteronism Type 1 with Identified ENaC Mutations (ENaC Group).

## Abbreviations

The following abbreviations are used in this manuscript:

PHA-1	Pseudohypoaldosteronism type 1
PHA-2	Pseudohypoaldosteronism type 2
MR	Mineralocorticoid receptor
ENaC	Epithelial sodium channel
Na	Sodium
K	Potassium
Cl	Chloride
NaCl	Sodium chloride
PRA	Plasma renin activity
ARC	Active renin concentration
ARR	Aldosterone/renin ratio
